# The role of primary cilia in congenital heart defect-associated neurological impairments

**DOI:** 10.3389/fgene.2024.1460228

**Published:** 2024-08-08

**Authors:** Nemanja Sarić, Nobuyuki Ishibashi

**Affiliations:** ^1^ Center for Neuroscience Research, Children’s National Medical Center, Washington, DC, United States; ^2^ Department of Pediatrics, Pharmacology and Physiology, George Washington University School of Medicine and Health Sciences, Washington, DC, United States; ^3^ Children’s National Heart Center, Children’s National Hospital, Washington, DC, United States

**Keywords:** congenital heart disease, genetic variants, cilia, brain, neurodevelopment

## Abstract

Congenital heart disease (CHD) has, despite significant improvements in patient survival, increasingly become associated with neurological deficits during infancy that persist into adulthood. These impairments afflict a wide range of behavioral domains including executive function, motor learning and coordination, social interaction, and language acquisition, reflecting alterations in multiple brain areas. In the past few decades, it has become clear that CHD is highly genetically heterogeneous, with large chromosomal aneuploidies and copy number variants (CNVs) as well as single nucleotide polymorphisms (SNPs) being implicated in CHD pathogenesis. Intriguingly, many of the identified loss-of-function genetic variants occur in genes important for primary cilia integrity and function, hinting at a key role for primary cilia in CHD. Here we review the current evidence for CHD primary cilia associated genetic variants, their independent functions during cardiac and brain development and their influence on behavior. We also highlight the role of environmental exposures in CHD, including stressors such as surgical factors and anesthesia, and how they might interact with ciliary genetic predispositions to determine the final neurodevelopmental outcome. The multifactorial nature of CHD and neurological impairments linked with it will, on one hand, likely necessitate therapeutic targeting of molecular pathways and neurobehavioral deficits shared by disparate forms of CHD. On the other hand, strategies for better CHD patient stratification based on genomic data, gestational and surgical history, and CHD complexity would allow for more precise therapeutic targeting of comorbid neurological deficits.

## 1 CHD-associated neurological and behavioral impairments

Children with CHD face the highest risk of infant mortality due to birth defects (Gilboa et al., 2010). Surgical technique advances have dramatically improved survival rates, however assessments of long-term outcomes in these patients have revealed a propensity for neurodevelopmental and neurobehavioral deficits. Cognitive delay, motor skill deficits, higher rates of autism spectrum disorder (ASD) and attention deficit hyperactivity disorder (ADHD) diagnoses as well as language difficulties represent the most frequently encountered neurological sequelae (Bellinger et al., 2003; Bellinger et al., 2009; Marelli et al., 2016; Morton et al., 2017). Longitudinal studies have found correlations between lower arterial oxygen saturation levels and worse motor skills in CHD patients, according to diagnostic criteria of the Bayley Scales of Infant Development III (Hoffman et al., 2016). In addition, subclinical perioperative seizures detected via electroencephalography (EEG) in a subset of CHD patients was predictive of executive function deficits, reduced sociability, and restrictive behaviors (Gaynor et al., 2013). Many of the impairments persist into late childhood and adolescence, manifesting as a lower intelligence quotient (IQ), worse scholastic performance, attenuated visuo-spatial and visuo-motor skills, and a greater frequency of behavioral issues (Bellinger et al., 2009). Neuroanatomically, the most consistent findings using magnetic resonance imaging (MRI) and diffusion tensor imaging (DTI) are white matter injury and immaturity (Gaynor, 2004; Beca et al., 2013), suggesting that CHD patients have impaired structural and functional connectivity. These clinical studies underscore the variety, yet specificity of detrimental neurobehavioral outcomes found among CHD patients, which likely involve multiple brain regions and circuits that are particularly vulnerable to CHD-linked risk factors.

## 2 Genetic risk factors

Evidence for a major role of genetic variants in CHD has mostly come from large-scale genomic and exome sequencing efforts, such as those performed by research groups of the Pediatric Cardiac Genomics Consortium (PCGC). These studies have focused on dissecting the genetic contribution to CHD cases presenting with or without neurodevelopmental deficits using parent offspring trios (Homsy et al., 2015). Many of the identified gene hits in the neurodevelopmental group were *de novo* loss of function variants which were associated with known genetic syndromes, with some genes also being linked to isolated CHDs.

### 2.1 Key genetic variants

Among the genes with damaging (premature truncation, frame shift or splice site mutations) *de novo* variants identified as significantly associated with CHD, key ontological categories include transcriptional regulation, morphogenesis, cilia formation, chromatin regulation and the connectome (Homsy et al., 2015; Ji et al., 2020). These variants were particularly enriched among CHD individuals with accompanying neurodevelopmental deficits in contrast to those without extra-cardiac anomalies. Many of these genes were also demonstrated to have high levels of expression in both the heart and brain. Independent CHD association studies have found multiple genomic hotspots for copy number variants (CNVs) with known pleiotropic and dosage-sensitive effects on cardiac and brain development, whose contribution is estimated at 10%–15% of CHD cases (Ehrlich and Prakash, 2022; Landis et al., 2023). Single nucleotide polymorphisms (SNPs) conferring elevated risk of CHD have also been detected in individual genomic loci (Wang F. et al., 2016). Alongside single gene alterations chromosomal aneuploidies such as trisomy 21/Down syndrome are well known for frequently manifesting CHD (Dimopoulos et al., 2023). Collectively these studies illustrate the existence of extensive genetic heterogeneity among CHD patients with comorbid neurological impairments, some of which is shared with equally multifactorial neurodevelopmental disorders.

### 2.2 Preponderance of cilia-related damaging variants in CHD

**FIGURE 1:**
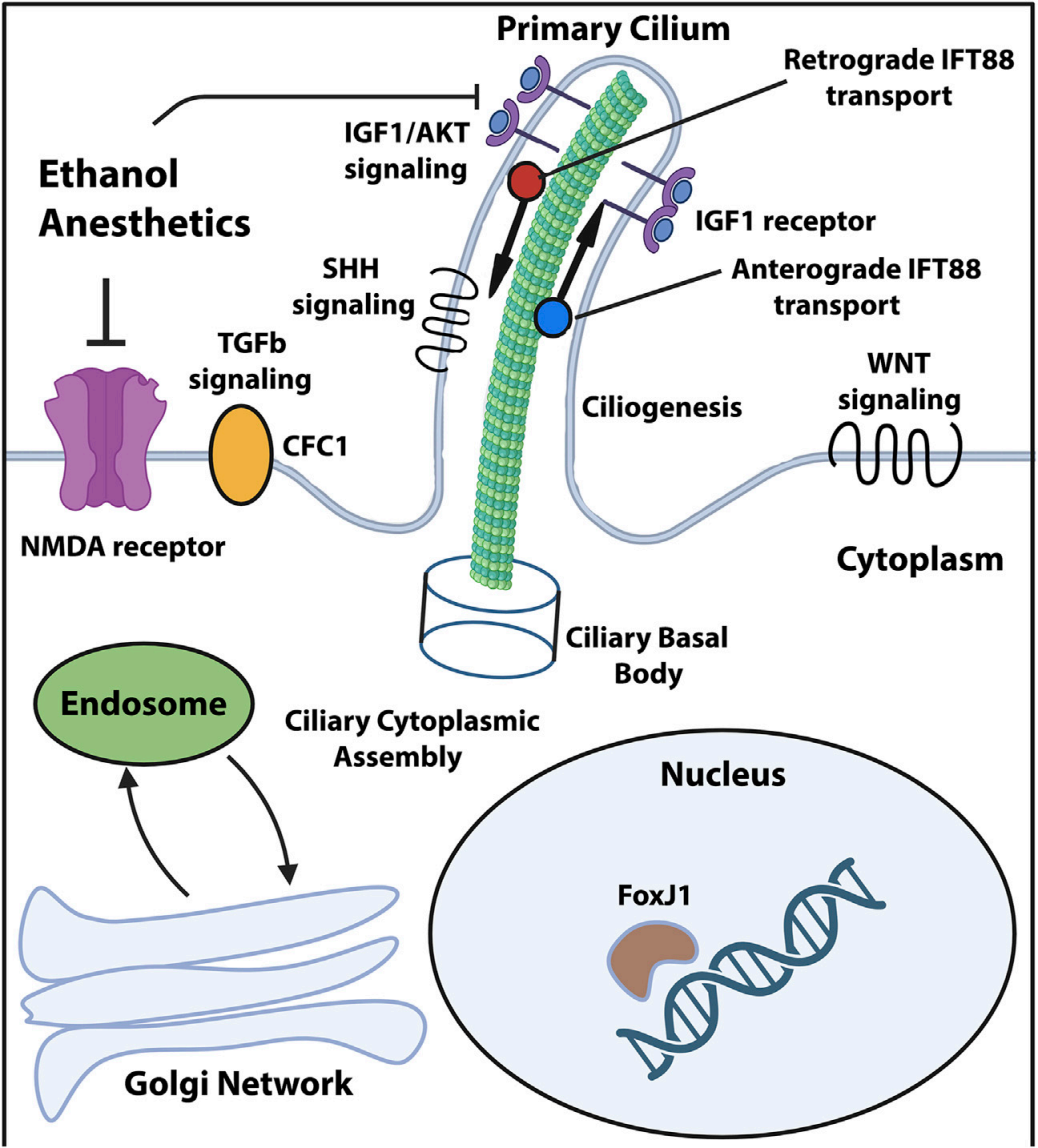
Ciliary compartments and pathways targeted by the known CHD mutations and environmental stressors. Diagram showing primary cilium and its compartments along with signaling pathways known to be affected by damaging genomic variants and environmental stressors associated with CHD and neurological impairments. Created in Biorender and adapted from Valente et al. (2014).

One of the most striking findings of the large-scale sequencing and forward genetic screening studies in CHD has been the extensive genetic contribution of cilia-associated variants. Loss of function mutations in genes such as *Foxj1*, *Cep110*, *Jbts17* and *Fuz* and their association with ciliary defects point to a central role for cilia signaling in CHD pathogenesis (Zaidi et al., 2013; Li et al., 2015). The uncovered variants cluster into categories related to ciliogenesis, endocytic function and cilia-transduced intracellular signaling (Figure 1). These findings suggest that disruption of multiple processes upstream and downstream of normal cilia function enhances the likelihood of CHD-linked cardiac malformations. In addition, since many of the same genes are required for appropriate neural development and brain maturation, it is conceivable that the same damaging mutations affect both the developing heart and brain in CHD patients with neurological impairments. For instance, loss of function of the *Foxj1* gene, encoding a transcription factor, was demonstrated to detrimentally impact murine postnatal ependymal cell differentiation in the neurogenic niche of the lateral ventricles (Jacquet et al., 2009) as well as postnatal olfactory bulb neurogenesis (Jacquet et al., 2011). In addition, *Foxj1* targeted mutations elicit reductions in length and numbers of motile cilia in mice and zebrafish, while patient heterozygous *FOXJ1* mutations cause ciliopathies associated with situs inversus and isolated CHD (Padua et al., 2023). Ciliopathies such as Joubert and Meckel syndromes are frequently associated with neurological defects (Valente et al., 2014) including hydrocephalus, corpus callosum hypoplasia, ataxia and intellectual disability. Since cilia can be both motile and immotile/primary, the functional consequences on cardiac and neural development might differ substantially, depending on the nature and origin of the ciliary insult.

## 3 Role for primary cilia

Primary cilia are organelles consisting of microtubular filaments which form predominantly singular protrusions from the cell membrane (Goetz and Anderson, 2010). The initial putative links between CHD and ciliopathy came from observations of randomized left-right patterning of the developing heart due to loss of motile cilia and the resultant randomized morphogen flow in the extraembryonic fluid of the embryonic murine node organizer (Nonaka et al., 1998; Brennan et al., 2002). Ciliary mutations causing primary ciliary dyskinesia (PCD) result in heart laterality defects, including transposition of the great arteries (TGA) and double outlet right ventricle (DORV) (Desgrange et al., 2018). Further evidence for a ciliary role in CHD pathogenesis came from a large-scale forward mutagenesis screen carried out in murine fetuses using ethylnitrosourea (ENU) (Li et al., 2015). Interestingly, there was a clear observation of segregation between motile and immotile/primary cilia genes according to the presence of heart laterality defects, with a roughly equal split between motile and primary cilia genes within the laterality deficit group. Motile cilia function has been suggested to impact the developing brain by altering cerebrospinal fluid flow patterns and volume, leading to brain dysplasia (Panigrahy et al., 2016). Primary cilia have also been identified as key regulators of neural development, being required for developmental phases spanning early neurogenesis, appropriate expansion of different precursor cell classes and neuronal maturation (Youn and Han, 2018; Park et al., 2019).

### 3.1 Neurodevelopmental functions of primary cilia

Ciliary patterning of the embryonic forebrain is an important determinant of early dorsoventral and rostrocaudal division formation, as demonstrated by the marked telencephalic disorganization in hypomorphic intraflagellar transport 88 (*Ift88*) mutant mice (Willaredt et al., 2008). The *Ift88* mutants lack key microtubular components of the intraflagellar transport (IFT) system which serves to transport protein cargo bidirectionally along the cilium axoneme. Loss of primary cilia in neural progenitors of the early mouse embryo (earlier than embryonic day 9) results in increased immature progenitor proliferation (Wilson et al., 2012), which is largely attributable to dampened Gli3 signaling, itself a negative regulator of the Sonic hedgehog (SHH) pathway (Matissek and Elsawa, 2020). Later, mid-gestational, loss of primary cilia does not result in overt deficits in neocortical development (Tong et al., 2014), suggesting that their developmental function is critical specifically during early forebrain patterning. In contrast to murine corticogenesis, the human fetal neocortex has significantly higher basal levels of hedgehog signaling, rendering its expansion potentially more sensitive to ciliary disruptions (Wang L. et al., 2016). Individuals with hypoplastic left heart syndrome (HLHS) are indeed known to have a greater risk of microcephaly, cortical mantle immaturity and agenesis of the corpus callosum (Glauser et al., 1990), while also harboring ciliome-related loss of function variants (Yagi et al., 2018). On the other hand, conditional ablation of primary cilia in murine hippocampal and cerebellar precursors results in significant reductions in adult hippocampal neural stem cells (Breunig et al., 2008) and cerebellar hypoplasia (Spassky et al., 2008) respectively. These findings argue that a subset of the ciliary mutations recovered from CHD screens regulate key stages of early neurodevelopment of different brain regions and therefore might be responsible for more severe neurological outcomes in CHD patients.

### 3.2 Ciliary neuroprotection

Emerging pre-clinical evidence points to a neuroprotective function for primary cilia from environmental exposures. In a forebrain-specific model of ciliopathy acute perinatal exposure to ethanol led to neuronal caspase activation and subsequent dendritic degeneration of deep layer pyramidal neurons (Ishii et al., 2021). Intriguingly, the phenotype was solely observed when ethanol exposure was combined with *Ift88* inactivation-induced primary cilia loss, indicative of a gene-environment interaction. This effect was dependent on caspase 3-mediated cytoskeletal remodeling and was reversible through pharmacological activation of the insulin-like growth factor 1 receptor-protein kinase B (IGF1R-AKT) pathway, which is known for its growth-promoting and anti-apoptotic cellular functions (Hemmings and Restuccia, 2012). Curiously, this study did not find evidence for caspase-mediated apoptotic loss of neurons following ethanol, suggesting a non-apoptotic mechanism of action. Ethanol acts as both a facilitator of γ-amino butyric acid (GABAergic) and inhibitor of N-methyl-D-aspartate (NMDA) neurotransmission (Nagy, 2008), a property which classifies it as a CNS depressant and which is shared with most general anesthetics (Petrenko et al., 2014).

### 3.3 Ciliary dysfunction and CHD perioperative anesthesia

A significant fraction of CHD neonates (estimated at 25%) require heart surgery within their first year of life, necessitating the use of general anesthesia (Moller et al., 1994). Despite being essential for suppressing consciousness and pain management during surgeries, inhalable and injectable anesthetics have been scrutinized due to a plethora of pre-clinical evidence pointing to their potential for developmental neurotoxicity (Jevtovic-Todorovic et al., 2003; Yon et al., 2005). A recent clinical trial did not find significantly altered neurobehavioral outcomes in 5-year-old children undergoing a short 1-h course of general anesthesia (McCann et al., 2019), however it did not account for genetic status of the participants. This is of particular concern given that pre-existing genetic susceptibility in CHD infants, such as ciliary gene variants, might adversely interact with general anesthetic exposures as previously described (Saric et al., 2022). More in depth pre-clinical modeling as well as careful clinical studies which account for multiple relevant variables are needed to dissect the risk and mechanisms of genetic status and anesthesia interactions.

## 4 Diagnostic and therapeutic outlooks

The multitude of factors which can influence risk of CHD-relevant neurological impairments necessitates a combination of detailed diagnostic patient stratification and therapies targeted at each potential high-risk phase for neurological injury. Currently proposed CHD therapeutic strategies span both pre- and post-operative phases and include maternal oxygen supplementation, progesterone, tetrahydrobiopterin (BH4) as well as mesenchymal stromal cell (MSC) administration during surgery (Kobayashi et al., 2021). In addition to therapy, more precise diagnostic tools encompassing genomic data and developmental history are needed to identify CHD patients at high risk of comorbid neurological impairments, coupled with the appropriate, specific treatment regime. Given that primary cilia function constitutes a key node for damaging genetic variants, potential environmental stressors such as anesthesia and gene-environment interactions, future diagnostic tools will necessarily be more comprehensive to account for these factors. Ciliome-directed therapies will likely target ciliogenesis, signaling pathways such as Sonic hedgehog (SHH), Wingless (WNT), transforming growth factor β (TGFβ) and insulin growth factor (IGF), as well as ciliary microtubular transport (Figure 1).

## 5 Concluding remarks

Here we aimed to account for ciliary dysfunction as a potential key causative element in the adverse neurodevelopmental and behavioral sequelae commonly encountered in CHD patients. In conclusion, while many of the ciliary genetic and environmental risk factors associated with CHD can produce neurodevelopmental deficits in independent fashion, there is an increasing appreciation for their interactions which can act as strong phenotypic modifiers. Investigating how these interactions might induce or enhance neurological impairment will yield potential avenues for better therapeutic management.

## References

[B1] Beca J. Gunn J. K. Coleman L. Hope A. Reed P. W. Hunt R. W. (2013). New white matter brain injury after infant heart surgery is associated with diagnostic group and the use of circulatory arrest. Circulation 127, 971–979. 10.1161/CIRCULATIONAHA.112.001089 23371931

[B2] Bellinger D. C. Newburger J. W. Wypij D. Kuban K. C. Duplesssis A. J. Rappaport L. A. (2009). Behaviour at eight years in children with surgically corrected transposition: the Boston Circulatory Arrest Trial. Cardiol. Young 19, 86–97. 10.1017/S1047951108003454 19079812 PMC4942187

[B3] Bellinger D. C. Wypij D. Duplessis A. J. Rappaport L. A. Jonas R. A. Wernovsky G. (2003). Neurodevelopmental status at eight years in children with dextro-transposition of the great arteries: the Boston Circulatory Arrest Trial. J. Thorac. Cardiovasc Surg. 126, 1385–1396. 10.1016/s0022-5223(03)00711-6 14666010

[B4] Brennan J. Norris D. P. Robertson E. J. (2002). Nodal activity in the node governs left-right asymmetry. Genes Dev. 16, 2339–2344. 10.1101/gad.1016202 12231623 PMC187443

[B5] Breunig J. J. Sarkisian M. R. Arellano J. I. Morozov Y. M. Ayoub A. E. Sojitra S. (2008). Primary cilia regulate hippocampal neurogenesis by mediating sonic hedgehog signaling. Proc. Natl. Acad. Sci. U. S. A. 105, 13127–13132. 10.1073/pnas.0804558105 18728187 PMC2529104

[B6] Desgrange A. Le Garrec J. F. Meilhac S. M. (2018). Left-right asymmetry in heart development and disease: forming the right loop. Development 145, dev162776. 10.1242/dev.162776 30467108

[B7] Dimopoulos K. Constantine A. Clift P. Condliffe R. Moledina S. Jansen K. (2023). Cardiovascular complications of down syndrome: scoping review and expert consensus. Circulation 147, 425–441. 10.1161/CIRCULATIONAHA.122.059706 36716257 PMC9977420

[B8] Ehrlich L. Prakash S. K. (2022). Copy-number variation in congenital heart disease. Curr. Opin. Genet. Dev. 77, 101986. 10.1016/j.gde.2022.101986 36202051

[B9] Gaynor J. W. (2004). Periventricular leukomalacia following neonatal and infant cardiac surgery. Semin. Thorac. Cardiovasc Surg. Pediatr. Card. Surg. Annu. 7, 133–140. 10.1053/j.pcsu.2004.02.007 15283363

[B10] Gaynor J. W. Jarvik G. P. Gerdes M. Kim D. S. Rajagopalan R. Bernbaum J. (2013). Postoperative electroencephalographic seizures are associated with deficits in executive function and social behaviors at 4 years of age following cardiac surgery in infancy. J. Thorac. Cardiovasc Surg. 146, 132–137. 10.1016/j.jtcvs.2013.04.002 23768805 PMC4617776

[B11] Gilboa S. M. Salemi J. L. Nembhard W. N. Fixler D. E. Correa A. (2010). Mortality resulting from congenital heart disease among children and adults in the United States, 1999 to 2006. Circulation 122, 2254–2263. 10.1161/CIRCULATIONAHA.110.947002 21098447 PMC4911018

[B12] Glauser T. A. Rorke L. B. Weinberg P. M. Clancy R. R. (1990). Congenital brain anomalies associated with the hypoplastic left heart syndrome. Pediatrics 85, 984–990. 10.1542/peds.85.6.984 2339047

[B13] Goetz S. C. Anderson K. V. (2010). The primary cilium: a signalling centre during vertebrate development. Nat. Rev. Genet. 11, 331–344. 10.1038/nrg2774 20395968 PMC3121168

[B14] Hemmings B. A. Restuccia D. F. (2012). PI3K-PKB/Akt pathway. Cold Spring Harb. Perspect. Biol. 4, a011189. 10.1101/cshperspect.a011189 22952397 PMC3428770

[B15] Hoffman G. M. Brosig C. L. Bear L. M. Tweddell J. S. Mussatto K. A. (2016). Effect of intercurrent operation and cerebral oxygenation on developmental trajectory in congenital heart disease. Ann. Thorac. Surg. 101, 708–716. 10.1016/j.athoracsur.2015.08.059 26542436

[B16] Homsy J. Zaidi S. Shen Y. Ware J. S. Samocha K. E. Karczewski K. J. (2015). *De novo* mutations in congenital heart disease with neurodevelopmental and other congenital anomalies. Science 350, 1262–1266. 10.1126/science.aac9396 26785492 PMC4890146

[B17] Ishii S. Sasaki T. Mohammad S. Hwang H. Tomy E. Somaa F. (2021). Primary cilia safeguard cortical neurons in neonatal mouse forebrain from environmental stress-induced dendritic degeneration. Proc. Natl. Acad. Sci. U. S. A. 118, e2012482118. 10.1073/pnas.2012482118 33443207 PMC7817201

[B18] Jacquet B. V. Muthusamy N. Sommerville L. J. Xiao G. Liang H. Zhang Y. (2011). Specification of a Foxj1-dependent lineage in the forebrain is required for embryonic-to-postnatal transition of neurogenesis in the olfactory bulb. J. Neurosci. 31, 9368–9382. 10.1523/JNEUROSCI.0171-11.2011 21697387 PMC3145804

[B19] Jacquet B. V. Salinas-Mondragon R. Liang H. Therit B. Buie J. D. Dykstra M. (2009). FoxJ1-dependent gene expression is required for differentiation of radial glia into ependymal cells and a subset of astrocytes in the postnatal brain. Development 136, 4021–4031. 10.1242/dev.041129 19906869 PMC3118431

[B20] Jevtovic-Todorovic V. Hartman R. E. Izumi Y. Benshoff N. D. Dikranian K. Zorumski C. F. (2003). Early exposure to common anesthetic agents causes widespread neurodegeneration in the developing rat brain and persistent learning deficits. J. Neurosci. 23, 876–882. 10.1523/JNEUROSCI.23-03-00876.2003 12574416 PMC6741934

[B21] Ji W. Ferdman D. Copel J. Scheinost D. Shabanova V. Brueckner M. (2020). *De novo* damaging variants associated with congenital heart diseases contribute to the connectome. Sci. Rep. 10, 7046. 10.1038/s41598-020-63928-2 32341405 PMC7184603

[B22] Kobayashi K. Liu C. Jonas R. A. Ishibashi N. (2021). The current status of neuroprotection in congenital heart disease. Child. (Basel) 8, 1116. 10.3390/children8121116 PMC8700367 34943311

[B23] Landis B. J. Helvaty L. R. Geddes G. C. Lin J. I. Yatsenko S. A. Lo C. W. (2023). A multicenter analysis of abnormal chromosomal microarray findings in congenital heart disease. J. Am. Heart Assoc. 12, e029340. 10.1161/JAHA.123.029340 37681527 PMC10547279

[B24] Li Y. Klena N. T. Gabriel G. C. Liu X. Kim A. J. Lemke K. (2015). Global genetic analysis in mice unveils central role for cilia in congenital heart disease. Nature 521, 520–524. 10.1038/nature14269 25807483 PMC4617540

[B25] Marelli A. Miller S. P. Marino B. S. Jefferson A. L. Newburger J. W. (2016). Brain in congenital heart disease across the lifespan: the cumulative burden of injury. Circulation 133, 1951–1962. 10.1161/CIRCULATIONAHA.115.019881 27185022 PMC5519142

[B26] Matissek S. J. Elsawa S. F. (2020). GLI3: a mediator of genetic diseases, development and cancer. Cell Commun. Signal 18, 54. 10.1186/s12964-020-00540-x 32245491 PMC7119169

[B27] Mccann M. E. De Graaff J. C. Dorris L. Disma N. Withington D. Bell G. (2019). Neurodevelopmental outcome at 5 years of age after general anaesthesia or awake-regional anaesthesia in infancy (GAS): an international, multicentre, randomised, controlled equivalence trial. Lancet 393, 664–677. 10.1016/S0140-6736(18)32485-1 30782342 PMC6500739

[B28] Moller J. H. Taubert K. A. Allen H. D. Clark E. B. Lauer R. M. (1994). Cardiovascular health and disease in children: current status. A special writing group from the task force on children and youth, American heart association. Circulation 89, 923–930. 10.1161/01.cir.89.2.923 8313589

[B29] Morton P. D. Ishibashi N. Jonas R. A. (2017). Neurodevelopmental abnormalities and congenital heart disease: insights into altered brain maturation. Circ. Res. 120, 960–977. 10.1161/CIRCRESAHA.116.309048 28302742 PMC5409515

[B30] Nagy J. (2008). Alcohol related changes in regulation of NMDA receptor functions. Curr. Neuropharmacol. 6, 39–54. 10.2174/157015908783769662 19305787 PMC2645546

[B31] Nonaka S. Tanaka Y. Okada Y. Takeda S. Harada A. Kanai Y. (1998). Randomization of left-right asymmetry due to loss of nodal cilia generating leftward flow of extraembryonic fluid in mice lacking KIF3B motor protein. Cell 95, 829–837. 10.1016/s0092-8674(00)81705-5 9865700

[B32] Padua M. B. Helm B. M. Wells J. R. Smith A. M. Bellchambers H. M. Sridhar A. (2023). Congenital heart defects caused by FOXJ1. Hum. Mol. Genet. 32, 2335–2346. 10.1093/hmg/ddad065 37158461 PMC10321388

[B33] Panigrahy A. Lee V. Ceschin R. Zuccoli G. Beluk N. Khalifa O. (2016). Brain dysplasia associated with ciliary dysfunction in infants with congenital heart disease. J. Pediatr. 178, 141–148. 10.1016/j.jpeds.2016.07.041 27574995 PMC5085835

[B34] Park S. M. Jang H. J. Lee J. H. (2019). Roles of primary cilia in the developing brain. Front. Cell Neurosci. 13, 218. 10.3389/fncel.2019.00218 31139054 PMC6527876

[B35] Petrenko A. B. Yamakura T. Sakimura K. Baba H. (2014). Defining the role of NMDA receptors in anesthesia: are we there yet? Eur. J. Pharmacol. 723, 29–37. 10.1016/j.ejphar.2013.11.039 24333550

[B36] Saric N. Hashimoto-Torii K. Jevtovic-Todorovic V. Ishibashi N. (2022). Nonapoptotic caspases in neural development and in anesthesia-induced neurotoxicity. Trends Neurosci. 45, 446–458. 10.1016/j.tins.2022.03.007 35491256 PMC9117442

[B37] Spassky N. Han Y. G. Aguilar A. Strehl L. Besse L. Laclef C. (2008). Primary cilia are required for cerebellar development and Shh-dependent expansion of progenitor pool. Dev. Biol. 317, 246–259. 10.1016/j.ydbio.2008.02.026 18353302 PMC4043448

[B38] Tong C. K. Han Y. G. Shah J. K. Obernier K. Guinto C. D. Alvarez-Buylla A. (2014). Primary cilia are required in a unique subpopulation of neural progenitors. Proc. Natl. Acad. Sci. U. S. A. 111, 12438–12443. 10.1073/pnas.1321425111 25114218 PMC4151724

[B39] Valente E. M. Rosti R. O. Gibbs E. Gleeson J. G. (2014). Primary cilia in neurodevelopmental disorders. Nat. Rev. Neurol. 10, 27–36. 10.1038/nrneurol.2013.247 24296655 PMC3989897

[B40] Wang F. Wang H. Wang L. Zhou S. Chang M. Zhou J. (2016a). Association between single nucleotide polymorphisms in NFATC1 signaling pathway genes and susceptibility to congenital heart disease in the Chinese population. Pediatr. Cardiol. 37, 1548–1561. 10.1007/s00246-016-1469-5 27567908

[B41] Wang L. Hou S. Han Y. G. (2016b). Hedgehog signaling promotes basal progenitor expansion and the growth and folding of the neocortex. Nat. Neurosci. 19, 888–896. 10.1038/nn.4307 27214567 PMC4925239

[B42] Willaredt M. A. Hasenpusch-Theil K. Gardner H. A. Kitanovic I. Hirschfeld-Warneken V. C. Gojak C. P. (2008). A crucial role for primary cilia in cortical morphogenesis. J. Neurosci. 28, 12887–12900. 10.1523/JNEUROSCI.2084-08.2008 19036983 PMC6671792

[B43] Wilson S. L. Wilson J. P. Wang C. Wang B. Mcconnell S. K. (2012). Primary cilia and Gli3 activity regulate cerebral cortical size. Dev. Neurobiol. 72, 1196–1212. 10.1002/dneu.20985 21976438 PMC3350755

[B44] Yagi H. Liu X. Gabriel G. C. Wu Y. Peterson K. Murray S. A. (2018). The genetic landscape of hypoplastic left heart syndrome. Pediatr. Cardiol. 39, 1069–1081. 10.1007/s00246-018-1861-4 29569026 PMC8565805

[B45] Yon J. H. Daniel-Johnson J. Carter L. B. Jevtovic-Todorovic V. (2005). Anesthesia induces neuronal cell death in the developing rat brain via the intrinsic and extrinsic apoptotic pathways. Neuroscience 135, 815–827. 10.1016/j.neuroscience.2005.03.064 16154281

[B46] Youn Y. H. Han Y. G. (2018). Primary cilia in brain development and diseases. Am. J. Pathol. 188, 11–22. 10.1016/j.ajpath.2017.08.031 29030052 PMC5745523

[B47] Zaidi S. Choi M. Wakimoto H. Ma L. Jiang J. Overton J. D. (2013). *De novo* mutations in histone-modifying genes in congenital heart disease. Nature 498, 220–223. 10.1038/nature12141 23665959 PMC3706629

